# Application of Laser-Induced Breakdown Spectroscopy in Detection of Cadmium Content in Rice Stems

**DOI:** 10.3389/fpls.2020.599616

**Published:** 2020-12-18

**Authors:** Wei Wang, Wenwen Kong, Tingting Shen, Zun Man, Wenjing Zhu, Yong He, Fei Liu, Yufei Liu

**Affiliations:** ^1^College of Biosystems Engineering and Food Science, Zhejiang University, Hangzhou, China; ^2^School of Information Engineering, Zhejiang A & F University, Hangzhou, China; ^3^School of Agricultural Equipment Engineering, Jiangsu University, Zhenjiang, China; ^4^Key Laboratory of Spectroscopy Sensing, Ministry of Agriculture and Rural Affairs, Hangzhou, China

**Keywords:** rice stem, cadmium, laser induced breakdown spectroscopy, chemometrics method, biomass resource

## Abstract

The presence of cadmium in rice stems is a limiting factor that restricts its function as biomass. In order to prevent potential risks of heavy metals in rice straws, this study introduced a fast detection method of cadmium in rice stems based on laser induced breakdown spectroscopy (LIBS) and chemometrics. The wavelet transform (WT), area normalization and median absolute deviation (MAD) were used to preprocess raw spectra to improve spectral stability. Principal component analysis (PCA) was used for cluster analysis. The classification models were established to distinguish cadmium stress degree of stems, of which extreme learning machine (ELM) had the best effect, with 91.11% of calibration accuracy and 93.33% of prediction accuracy. In addition, multivariate models were established for quantitative detection of cadmium. It can be found that ELM model had the best prediction effects with prediction correlation coefficient of 0.995. The results show that LIBS provides an effective method for detection of cadmium in rice stems. The combination of LIBS technology and chemometrics can quickly detect the presence of cadmium in rice stems, and accurately realize qualitative and quantitative analysis of cadmium, which could be of great significance to promote the development of new energy industry.

## Introduction

Rice straw is an important secondary product of rice (Sepaskhah and Yousofi-Falakdehi, 2009; Zaima et al., 2010). For every kilogram of grain harvested, the yield of straw will increase by 1.0–1.5 kilograms. Straw has the advantages of abundant raw materials, low price, high combustion calorific value (about 50% of standard coal) (Jenkins et al., 1998; Lim et al., 2012; Yin et al., 2013). It is rich in nutrients such as mineral elements (N, P, K, Ca, Mg, and Si), plant fiber (cellulose, hemicellulose, and lignin) and protein, which can be used as fuel, feed, fertilizer, base material and industrial raw materials (Buzarovska et al., 2008; Abraham et al., 2016; Ostos-Garrido et al., 2019; Logeswaran et al., 2020). The scientific use of straw can not only effectively alleviate the current situation of energy supply shortage, but also solve environmental pollution problems (Matsumura et al., 2005; Hernández et al., 2019). However, rice plants have strong absorption properties for cadmium (Liu et al., 2019). When environment is contaminated with heavy metals, it will harm growth of plants and accumulate in the plants (Xue et al., 2017; Cheng et al., 2018; Han et al., 2018). Studies have shown that in the cadmium absorption process of rice, cadmium is more easily transferred from roots into stem (Xie et al., 2015). Most of the cadmium absorbed by rice roots is concentrated in the stem, which is an important factor causing heavy metal pollution of straw biomass resources. Previous studies have shown that the presence of cadmium can affect the performance of straw as biomass (Narodoslawsky and Obernberger, 1996; Kirkelund et al., 2013; Yang et al., 2015). During the anaerobic digestion of straw, cadmium will be released into biogas slurry, which inhibits microbial activity and thus hinders the biogas production (Nzihou and Stanmore, 2013). During the combustion of biomass, organic matter is decomposed, while heavy metals only be partially or fully gasified at high temperatures, accumulated in ash residue or released into the environment with flying dust, forming secondary pollution (Fernandez et al., 1992; Lu et al., 2012; Delplanque et al., 2013; Sánchez et al., 2015). The presence of heavy metals in straw is likely to be a factor limiting the function as biomass (Sas-Nowosielska et al., 2004; Laval-Gilly et al., 2017). Therefore, rapid detection of cadmium in rice straw is of great significance to promote the development of straw industry and new energy industry.

Commonly used heavy metal detection methods include atomic absorption spectroscopy (AAS), atomic fluorescence spectroscopy (AFS), X-ray fluorescence spectroscopy (XRFS), and inductively coupled plasma emission spectroscopy (ICP-OES) (Fortes et al., 2013; Kim et al., 2013). Traditional heavy metal methods require sampling, pretreatment and laboratory chemical analysis, which are tedious, costly and time-consuming (Rai and Rai, 2008; Gondal et al., 2010). Laser induced breakdown spectroscopy (LIBS) has been widely applied in the fields of solid, liquid and gas as a new method of material element analysis since it was proposed in 1962 (Kim et al., 2013; van Maarschalkerweerd and Husted, 2015). LIBS can quickly obtain information on sample composition and elements content in a short time. Compared with other detection technologies, LIBS has many advantages, such as less sample required, no complex pretreatment, multi-element joint measurement, and fast implementation (Gondal et al., 2009). At present, some studies have focused on the application of LIBS to detect pollution of heavy metals. Zhao et al. (2019) studied the quantitative analysis of Pb in soil and demonstrated that dual-pulse laser induced breakdown spectroscopy (DP-LIBS) can be applied as an efficient spectroscopic tool to improve the quantitative analysis of Pb heavy metal in soil. Rehan et al. (2020) applied LIBS to estimate the amount of toxic heavy metals (Pb, Cr, Ni) in different brands of face foundation powders. Peng et al. (2019a) used collinear DP-LIBS to achieve determination of chromium content in rice leaves. However, as far as we know, LIBS has not been used to detect heavy metals in rice straws. Therefore, the detection of heavy metals in straw based on LIBS technology is of great significance for the utilization of straw as a biomass.

Based on the purpose of preventing potential risk of heavy metals in biomass, this paper selected rice stem as the research object, and introduced a rapid detection method of cadmium in rice stem of LIBS technology. The specific objectives of this paper are as follows: (1) to improve spectral stability with pretreatment methods of wavelet transform (WT) and median absolute deviation (MAD); (2) to visualize the distribution of stems with different cadmium by principal component analysis (PCA); (3) to establish classification models for fast discrimination of cadmium stress degree in rice stems; and (4) to establish multivariate models for quantitative detection of cadmium in rice stems.

## Materials and Methods

### Materials

In the experiment, 10 pots of rice were cultivated to obtain rice stems with different cadmium concentrations. The rice variety selected in the experiment was Xiushui 134, which was a single-season conventional late japonica rice widely cultivated in Zhejiang Province, China. During rice cultivation, the international rice nutrient solution formula was adopted, and the pH value was set as 5.3–5.6. The rice was placed in an artificial climate chamber. The cultivation parameters were as follows: the duration of day mode was 14 h, the temperature was 30°C, the relative humidity was 85%, and the light intensity was 225 μmol⋅m^–2^⋅s^–1^; the duration of night mode was 10 h, the temperature was 22°C, and the relative humidity was 85%. When entering the tillering stage, 10 pots of rice were divided into five groups of two pots each. CdCl_2_ was used to prepare cadmium solutions with different concentrations. Based on references and experimental experience, Cd^2+^ concentrations were, respectively, set to 0 (CK), 5, 25, 50, and 100 μM. CdCl_2_ solution with corresponding concentration was added to nutrient solution (Peng et al., 2019b).

Rice plants with similar growth were selected from each group, at 10, 20, and 30 days under heavy metal, and stems were collected as samples. A total of 15 rice stem samples with different cadmium concentrations were obtained, namely Day 10-CK, Day 10–5, Day 10–25, Day 10–50, Day 10–100, Day 20–CK, Day 20–5, Day 20–25, Day 20–50, Day 20–100, Day 30-CK, Day 30–5, Day 30–25, Day 30–50, and Day 30–100. To remove impurities and metal ions attached to the surface of sample, washed the collected sample several times with distilled water, then immersed with EDTA-2Na solution for about 60 min, and finally washed again with distilled water. Drained the water, and dried the sample in a 60°C oven for a period of time to constant weight, which was about 72 h. Put the dried stems into 5 ml centrifuge tube and add five grinding beads with the diameter of 2.8 mm. Then, put the above centrifuge tube into the grinding tool, and use a grinder (JXFSTPRP-48, Shanghaijingxin, Shanghai, China) to shake and grind for 2 min at a frequency of 60 Hz to obtain uniform stem powder. 200 mg of powder was weighed from each sample for tableting. The powder was pressed into a tablet of 10 mm × 10 mm × 1 mm by a tablet machine (YLJ-20T, Guoyan, Hebi, China) at a pressure of 60,000 N for 20 s. Four tablets were prepared from stems of each cadmium concentration to collect LIBS spectral data.

### Experimental Setup

**FIGURE 1:**
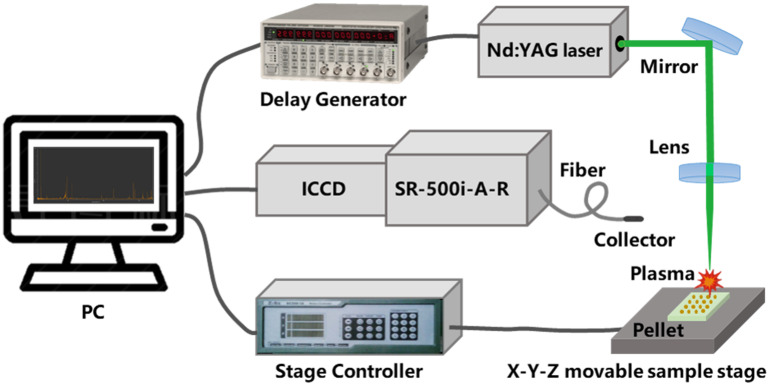
Structural diagram of LIBS equipment.

The LIBS equipment schematic diagram in the experiment is described in Figure 1. Q-switched Nd:YAG nanosecond pulsed solid-state laser (Vlite-200, Beamtech Optronics, Beijing, China) was applied as laser sources to produce laser pulses with a wavelength of 532 nm, an energy of 60 mJ, a pulse frequency of 1 Hz, a pulse width of 8 ns. The energy meter (StarLite, Ophir, Jerusalem, Israel) was applied to calibrate laser energy to ensure accuracy. In the optical path system, the laser pulses passed through half-wave plate, polaroid, mirrors and lens successively, and converged into a light spot with a diameter of 7 mm. To obtain better ablation, the sample position should be located 98 mm below focusing lens, where the focal length is 100 mm. Adopt the *X*–*Y*–*Z* movable sample stage (Zolix, Beijing, China) to realize real-time movement of samples. The laser pulses converge on the sample surface under the action of a lens, ablating the sample to generate plasma. Taking monochromator (SR-500i-A-R, Andor, Belfast, United Kingdom) as a spectrometer, which enable separate the optical signal during plasma transition process, so as to obtain high-resolution spectral information in the short wavelength band of 210.0107∼230.9990 nm. The wavelength of atomic and ion spectra is in one-to-one correspondence with specific elements, and the intensity of the spectral signal has a quantitative relationship with the content of the corresponding element (Rodrigues Romera et al., 2016). ICCD detector (iStar DH334T-18F-03, Andor, Belfast, United Kingdom) converted optical signal into electrical signal with optimized parameters (Delay = 1 μs, Gate width = 10 μs, Gain setting = 1000). The digital delay generator (DG645, Stanford Research Systems, California, United States) enables realize time series control between lasers and ICCD detectors. Digital delay generator has a delay range of 0–2,000 ns and a resolution of 5 ps. In order to obtain stable spectra, spectra were collected at 16 different locations on each sample. Use *X*–*Y*–*Z* movable sample stage to realize the path setting of 16 acquisition positions, in which the path is a square matrix with a side length of 6 mm. During the data acquisition process, each of the 16 locations was collected for 5 times and accumulated as spectral data, where the repetition frequency is 1 Hz, in an attempt to avoid spectral fluctuations. As a result, the spectral data of each rice stem was the average of 80 spectra at 16 locations. It takes less than a minute to collect 80 spectral data from a sample.

### Standard Method for Cadmium Content Determination

**FIGURE 2:**
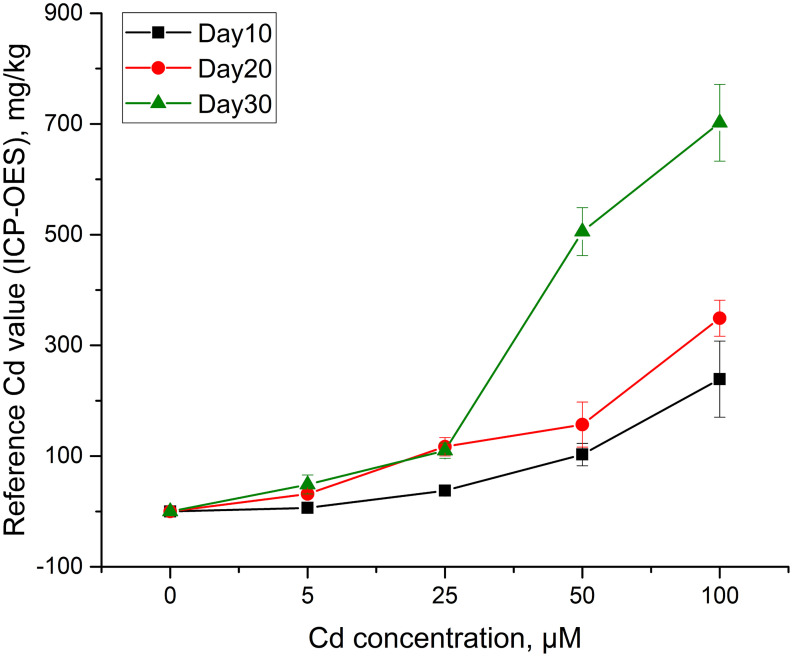
The reference content of cadmium in rice stem obtained by ICP-OES.

Taking inductively coupled plasma optical emission spectrometry (ICP-OES) as the standard method to detect the reference content of cadmium in rice stem. The stem tablets from which spectra have been collected were reground into powder. Weigh 100 mg samples from each tablet into the digestion tube, add 5 mL of nitric acid and 1 mL of hydrogen peroxide, tighten the digestion tube, and place them in a microwave digestion apparatus (MARS 6, CEM, Matthews, United States) for digestion. After digestion, transfer the digestive solution to a centrifuge tube, dilute with deionized water to the scale line, and shake it evenly. Finally, cadmium content of the solution was measured by ICP-OES (Optima 8000, PerkinElmer, Waltham, United States). In addition, the solution of standard sample of citrus leaves (GBW10020, Beijing, China) and blank sample were prepared simultaneously according to the above conditions. In the experiment, it took about 150 min to detect the cadmium content in a stem sample using ICP-OES. As described in Figure 2 that under the same time conditions, as the cadmium concentration increased, the average cadmium content in the corresponding group also increased successively. Among them, the cadmium content of two groups of stem samples was very close, which were group of Day 20–5 and Day 30–5, and group of Day 20–25 and Day 30–25, respectively. The average cadmium content of rice stems from Day 30–100 was 702.25 mg/kg, reaching the highest accumulation of cadmium under experimental conditions. ICP-OES detection value was used as a reference for fast detection of heavy metal content in quantitative analysis.

### Data Processing

**FIGURE 3:**
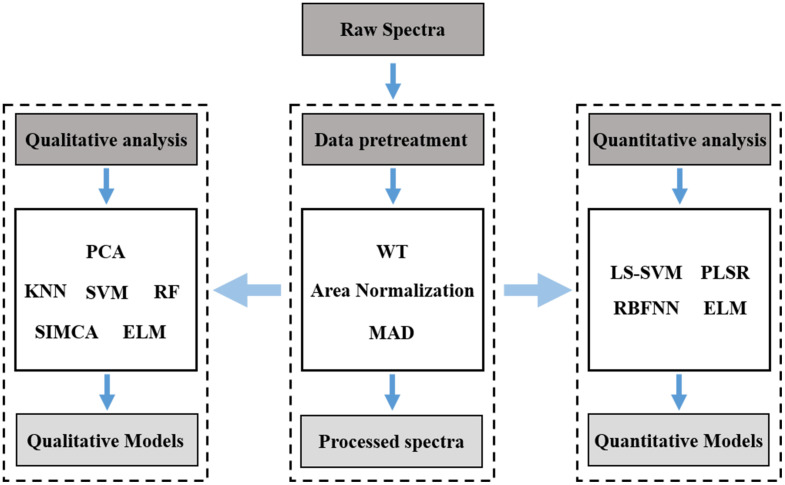
Data processing flow.

Rice stems are heterogeneous substance, and their spectra are extremely complex, containing a lot of redundant information (Lu et al., 2019). The LIBS detection of stem elements is usually accompanied with matrix effect (Markiewicz-Keszycka et al., 2017). To improve the analysis accuracy, it is necessary to adopt suitable methods to deal with the spectrum. The data processing process in this paper consists of data pretreatment, qualitative analysis and quantitative analysis, as described in Figure 3.

#### Data Pretreatment

During the laser ablation process, the parameters of the instrument and the physical characteristics of the sample itself will cause spectral fluctuations and outliers. Pretreatment methods were used to improve the stability of detection signal. WT was adopted to denoise LIBS spectral data, in which db4 of Daubechies wavelet was used to decompose signals in three layers. Area normalization was used to reduce point-to-point fluctuations between single spectra. The MAD was used to eliminate abnormal spectra due to the randomness of the plasma transition (Peng et al., 2019a).

#### Chemometrics Method

Chemometrics is a new branch of chemistry. It applies knowledge such as mathematics, statistics and computer to scientifically design experiments, select the optimal measurement method, obtain the most effective characteristic data, and extract the information about substances to the maximum extent (Zhang et al., 2018). Due to the influence of laser energy fluctuation, sample non-uniformity and matrix effects, complex LIBS spectra are generated. Considering the complexity of data, chemometrics methods are often used in combination with LIBS spectroscopy to improve the stability and reliability of real-time analysis (Lang et al., 2018). In order to accurately detect cadmium in rice stem, the chemometrics method and LIBS technology were combined for qualitative and quantitative analysis. In order to determine the optimal effect of discriminant analysis and quantitative detection, a variety of modeling methods were used for comparison in the paper.

Principal component analysis is a data dimension reduction method that converts all raw variables into a few unrelated variables. Each principal component is a linear combination of raw variables, which can reduce the complexity of the data and find the most useful functions, with low information loss (Zhan et al., 2015). When using PCA for qualitative analysis, calculate the contribution rate of principal components, and the distribution map of the principal component in the sample set is obtained for cluster analysis (Moncayo et al., 2017). K-nearest neighbours (KNN) is a pattern recognition algorithm (Godoi et al., 2011). The distance between the data to be tested and the data in the training set are sorted in increasing order. Then the *K* samples closest to the sample to be tested are selected to determine the frequency of occurrence of the category of the *K* samples. The category with the highest frequency among the samples is taken as the category of test data (Ghasemi-Varnamkhasti and Forina, 2014). Support vector machine (SVM) has advantages in solving the classification of small samples, non-linear and high-dimensional data, and it belongs to a supervised learning method (Fei, 2010). Due to the simplicity of RBF function and its ability to solve complex problems, RBF function was chosen as the kernel function of SVM model for classification. Soft independent modeling of class analogy (SIMCA) is a supervised pattern recognition method based on PCA (Basri et al., 2017). It establishes a PCA model for each sample category, and projects unknown samples according to each principal component model for discriminant analysis (Ye et al., 2008). SIMCA was used for classification of rice stems. Random forest can learn the mapping relationship between features and labels from the data, which belongs to the category of supervised learning. It is a kind of classifier composed of several decision trees, the output category of which depends on the category output mode of an individual tree, and it is a typical representative of the strong classifier composed of several weak classifiers (Xin et al., 2012). Extreme learning machine (ELM) is an effective learning algorithm for single-hidden layer feedforward neural network (SLFNN) (Feng et al., 2019). ELM can randomly initialize the input weight and biases of SLFNN and obtain the corresponding output weight (Chen et al., 2012). Traditional feedforward neural network takes a long time to train and often gets into the local minimum point, while ELM method can solve the above problems with its rapid learning speed and good generalization performance. We used ELM to distinguish the cadmium pollution and to detect the cadmium content. As an improved algorithm of SVM, least square support vector machine (LS-SVM) replaces the non-equal constraints of SVM optimization problem with equality constraints, transforms the solution of SVM into the solution of linear equations, which improves the efficiency and reduces the difficulty (Pierna et al., 2011). RBF function is used as the kernel function of LS-SVM, and two parameters, sig2 and gam, are involved in the model training process. Among them, sig2 is the parameter kernel function, here is the bandwidth in the case of RBF, and gam is the regularization parameter, which determines the trade-off between the minimum model complexity and the minimum training error. Partial least squares (PLS) provides a multivariable modeling method (Maquina et al., 2019). It can effectively deal with multicollinear problems and establish linear regression models. The main idea of partial least square method is to project the original variables into mutually orthogonal dimensions through linear variables, producing latent variable (LV) (Musingarabwi et al., 2016). In the PLS model, latent variables (LVs) can explain most of the variables in the sample. It is necessary to select the appropriate number of LVs to obtain the best effect. In this paper, cross-validation method was used to select the optimal number of LVs. In order to avoid overfitting, the maximum number of variables was determined in one-tenth of the total number of samples (Gowen et al., 2011; Goto et al., 2015). Using the measured values of the sample to assign values to the Y matrix, PLS can be used as partial least squares regression (PLSR) for regression analysis (Peng et al., 2019b). Radial basis function neural network (RBFNN) is a feedforward network with a single hidden layer based on function approximation, in which the first layer is the input layer, the second is the hidden layer, the third is the output layer (Feng et al., 2018). RBFNN has been widely used for its simple training structure, fast convergence speed, strong generalization ability and arbitrary approximation.

## Results and Discussion

### LIBS Spectral Analysis

**FIGURE 4:**
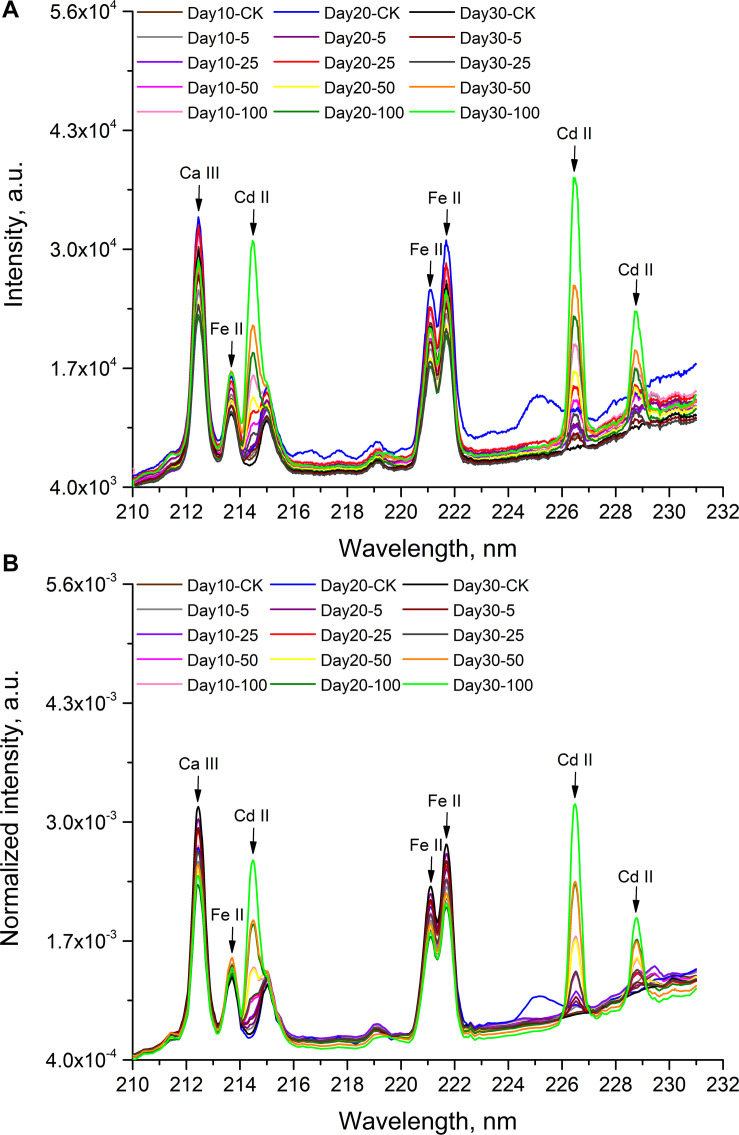
LIBS spectra of rice stem tablets with different Cd concentrations in the range of 210.01∼231.00 nm. **(A)** average raw spectra; **(B)** average spectra after pretreatment.

The representative LIBS spectra of rice stems under different cadmium stress are illustrated in Figure 4, where (A) is the average raw spectra and (B) is the average spectra after pretreatment. It can be seen from the Figure 4A that rice stems with different cadmium concentrations had similar emission lines, which are mainly related to organic compounds, nutrients and cadmium. The pretreatment methods were used to remove noise, fluctuations and outliers from the raw spectrum. In the spectral range of 210.01∼231.00 nm, three cadmium emission lines can be clearly observed with reference to the atomic spectra database of National Institute of Standards and Technology (NIST), namely ionic spectral lines Cd II 214.44 and Cd II 226.50 nm, and atomic spectral lines Cd I 228.80 nm. We also found that emission lines Ca III 212.30, Fe II 213.77, Fe II 221.10, and Fe II 221.71 nm emerged near the emission lines of cadmium. The spectral characteristics of Ca, Fe, and Cd in different samples are significantly different, as described in Figure 4B. In all samples, Day 30–100 had the highest spectral line intensity at three characteristic lines of cadmium including 214.44, 226.50, and 228.80 nm. It was consistent with the results in ICP-OES, which showed that the sample with the largest accumulation of cadmium was Day 30–100. Although it can be observed that cadmium-contaminated stem samples have different spectral line characteristics, it is not a straightforward process to complete accurate and rapid sample classification based on the spectral peak position or peak intensity of the elements. For specific elements, the characteristic spectral lines of the detected element may be different due to the matrix effect. In addition, the cadmium content of some samples from 15 different stems (Day 10–5, Day 20–5, and Day 30–5, Day 20–25 and Day 30–25) is very close, which will interfere with the classification effect. Multiple emission lines need to be analyzed in a more efficient way to accurately classify stem samples. Therefore, it was necessary to use chemometrics to find differences and achieve good identification.

### Cluster Analysis

**FIGURE 5:**
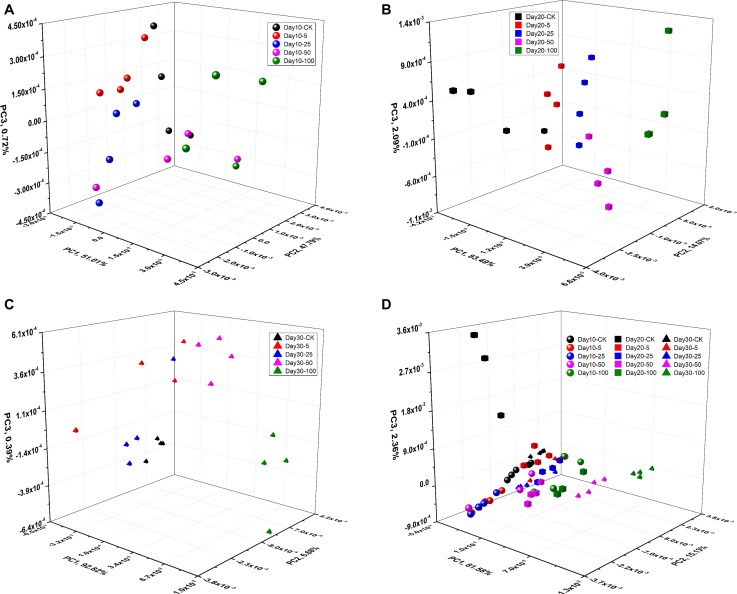
PC scores scatter plots of rice stem tablets. **(A)** PC1-PC2-PC3 for Day 10; **(B)** PC1-PC2-PC3 for Day 20; **(C)** PC1-PC2-PC3 for Day 30; **(D)** PC1-PC2-PC3 for all samples.

We used PCA to classify different stems, and visualize the distribution of them in the principal component (PC) scores scatter plots. PCA analysis was performed on the four group samples of Day 10, Day 20, and Day 30 and all samples (Day 10, Day 20, and Day 30). Figure 5 shows the PCA visualization of rice stems at CK, 5, 25, 50, and 100 μM concentrations under different stress days. Each point in the 3D scatter plot represented a sample, which can visually show the clustering effect. For the samples of Day 10, Day 20, and Day 30 and all samples, the total variance of the first three principal components was, respectively, 99.53, 99.66, 99.89, and 99.07%.

It can be seen that the clustering effects of rice stem scatters vary in different days. For Day 10 samples, five clusters were easily found in the PC1-PC2-PC3 space. However, the clustering effect of stems with different cadmium concentrations was different. As presented in Figure 5A, for CK, 5, 25 and 100 μM stem samples, each type tended to cluster together, which made these type of samples easier to distinguish from other samples. However, the 50 μM samples was close to the adjacent concentration gradient samples (25, 100 μM), which made it difficult to get good seperation between three concentration samples (25, 50, and 100 μM). For Day 20 and Day 30 samples, as depicted in Figures 5B,C, it was clearly that five clusters were found in the spaces. Rice stems were more likely to cluster together, under the same stress concentration. The five rice stem samples can be well separated from each other. In Figure 5C, a sample of 25 μM tended to cluster with 5 μM samples. For all the samples (Day 10, Day 20, and Day 30), PCA analysis was performed on fifteen different rice stems, as shown in Figure 5D. Fifteen clusters were obviously found in the spaces. However, the 15 types of samples tend to cluster together more than in previous cases. This is because under the combined effect of time and concentration, the cadmium content of two groups of stem samples was very close, which were Day 20–5 and Day 30–5, and Day 20–25 and Day 30–25, respectively. We can see from Figure 5D, 5 μM of 20 day and 5 μM of 30 day were clustered together, and 25 μM of 20 day and 25 μM of 30 day were also clustered, although the two did not belong to the same type of stress gradient.

In this paper, PCA was carried out on the stems of different rice stems, and sample scatter plots were made in three-dimensional space to visualize clustering effect of stems contaminated with different cadmium. For the stems of Day 10, Day 20, and Day 30, PCA analysis can achieve better clustering effect. But for all samples (Day 10, Day 20, and Day 30), 15 different stems cannot be distinguished well because samples of different gradients had similar cadmium concentrations. In addition, PCA is a clustering analysis method, which can clearly visualize the clustering effect of samples and cannot provide accurate discriminant results. Therefore, supervised multivariate analysis was used to predict contamination degree and content of cadmium in the next section.

### Rapid Discrimination of 15 Cadmium Stress

The spectra of different rice stems after pretreatment were used to establish classification models including KNN, SVM, SIMCA, RF, and ELM to predict cadmium content. Before modeling, sample division was performed. For every four samples, the first three were used for calibration and the remaining one for prediction. Thus, 45 tablets were selected to calibrate model and 15 tablets were used to predict sample properties, with a ratio of 3:1. For the discriminant analysis model, accuracy was used as a performance indicator to evaluate the model, that was, the proportion of the number of correctly classified samples to the total number of samples.

The discriminant models results of rice stems with 15 different cadmium concentrations are shown in the Table 1. The ability of different chemometrics models to discriminate the cadmium stress of stems was compared. The discrimination effect of KNN is poor, and the accuracy of the calibration set and the prediction set were 75.56 and 53.33%, respectively. Compared with KNN, the discriminative effect of SVM, SIMCA and RF has improved. The accuracy of calibration set was 91.11, 95.56, and 100%, respectively, and the accuracy of their prediction set was 73.33%. ELM has the best discriminating effect, with 91.11% of calibration accuracy and 93.33% of prediction accuracy. Comparing the ability of different models to discriminate the cadmium concentration of rice stems, we can find that ELM had the best effect, in which the accuracy of calibration and prediction were both more than 90%, followed by SVM, SIMCA and RF models, in which the calibration accuracy of models all exceeded 90% while the prediction efficiency was only 73.33%, and KNN had the worst discrimination effect, in which the accuracy of correction and prediction did not exceed 80%. Therefore, compared with other methods, ELM has higher discriminant accuracy, which is more advantageous in the discriminant analysis of cadmium pollution degree in rice stems. It takes no more than 5 min to classify a stem sample based on the above method. LIBS method can quickly distinguish 15 cadmium-contaminated stems. These results indicate that LIBS combined with ELM can quickly detect cadmium in stems, and accurately distinguish the degree of cadmium pollution. The combination of chemometrics and LIBS provides a fast method for discriminant analysis of cadmium content in rice stems.

**TABLE 1:** Results for cadmium stress discrimination in rice stems.

Model	Parameter^*a*^	Accuracy of calibration set	Accuracy of prediction set
KNN	3	75.56%	53.33%
SVM	(1.000, 1,000)	91.11%	73.33%
SIMCA	(2, 2, 2, 2, 2, 2, 2, 2, 2, 2, 2, 2, 2, 2, 2)	95.56%	73.33%
RF	(151,7)	100%	73.33%
ELM	12	91.11%	93.33%

*^a^Parameters of different models: the k-value for KNN, different penalty parameters (c) and kernel function parameters (g) for SVM, the number of principal components (PCs) of SIMCA, number of trees in the forest and nodes per tree for RF, and the number of hidden nodes of ELM.*

### Quantitative Detection of Cadmium Content

Multivariate models such as LS-SVM, PLSR, RBFNN, and ELM were established to complete the quantitative detection of cadmium in rice stems based on LIBS. The LIBS spectra ranged from 210.01 to 231.00 nm and contained 1,024 variables. 45 samples were selected as the calibration set, and 15 samples were used as the prediction set. Using correlation coefficient (R) and root mean square error (RMSE) as indicators to evaluate model performance. Multivariate models results of cadmium concentration in rice stems based on PLSR, LS-SVM, RBFNN, and ELM are shown in Table 2. Obviously, the four models all showed good results in predicting cadmium content in rice stems. For LS-SVM models, the optimal parameters were determined based on grid-search procedure, with sig2 and gam of 5.492 × 10^3^ and 3.712 × 10^3^. LS-SVM models achieved good performance, with *R*_*c*_ of 0.999, RMSEC of 9.42 mg/kg, *R*_*p*_ of 0.976 and RMSEP of 46.47 mg/kg. In PLSR modeling process, leave-one-out cross validation was used to select LVs to determine the model, and LVs was 4. *R*_*c*_ reached 0.972 and RMSEC was 47.80 mg/kg, *R*_*p*_ reached 0.991 and RMSEP was 35.60 mg/kg. The spread coefficient of RBFNN was optimized, which ranges from 1 to 1000, with a step size of 1. The number of hidden nodes of ELM was optimized, ranging from 1 to 45, with a step size of 1. Compared with LS-SVM and PLSR, RBFNN and ELM had better prediction effects, in which *R*_*c*_ and *R*_*p*_ of the above two models both exceeded 0.98.

**TABLE 2:** Multivariate models results for cadmium concentrations in stems.

Model	Parameter^*a*^	Accuracy of calibration set		Accuracy of prediction set	
		*R*_*c*_	RMSEC (mg/kg)	*R*_*p*_	RMSEP (mg/kg)
LS-SVM	(5.492 × 10^3^, 3.712 × 10^3^)	0.999	9.42	0.976	46.47
PLSR	4	0.972	47.80	0.991	35.60
RBFNN	938	0.985	35.31	0.990	37.05
ELM	11	0.981	39.80	0.995	28.96

*^a^Parameters for models: the bandwidth of kernel function (sig2) and the trade-off between the minimum model complexity and the minimum training error (gam) of LS-SVM, optimal number of latent variables (LVs) of PLSR, spread coefficient of RBFNN, the number of hidden nodes of ELM.*

**FIGURE 6:**
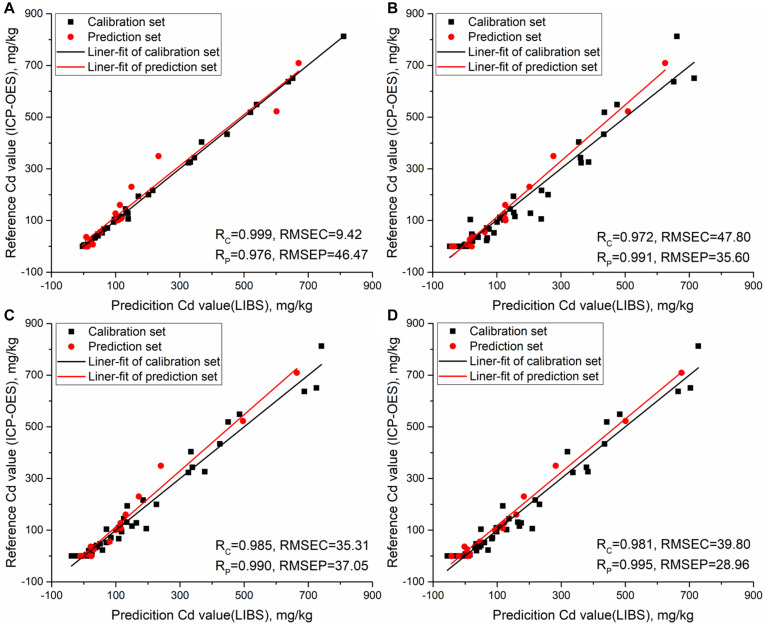
Relationship between reference cadmium value and prediction cadmium value based on LIBS in rice stems under different models. **(A)** LS-SVM model; **(B)** PLSR model; **(C)** RBFNN model; **(D)** ELM model.

Figure 6 shows the relationship between reference cadmium value and prediction cadmium value based on LIBS in rice stems under different models. We can observe that the data of calibration and prediction set in the four models are well fitted. For ELM, the model had the best correlation and lowest prediction error, where *R*_*p*_ is 0.995 and the RMSEP is 28.96 mg/kg in prediction set. The quantitative determination of cadmium in a stem sample takes no more than 5 min. The results showed that multivariate analysis with ELM method can realize the fast detection of cadmium in stem more effectively.

## Conclusion

In this paper, the combination of LIBS and chemometrics method successfully achieved the discriminant analysis and quantitative detection of rice stems with different cadmium concentrations. With the help of WT, area normalization and MAD, the noise, fluctuation and outliers in the raw spectrum were improved. PCA method was applied for cluster analysis of 15 different cadmium stresses rice stems, and it visualized the distribution of different samples in scores scatter plots. The classification models including KNN, SVM, SIMCA, RF, and ELM were established to distinguish stems with different cadmium stress degree. Compared to other models, ELM had the best discriminating effect, with 91.11% of calibration accuracy and 93.33% of prediction accuracy. The results indicate that LIBS combined with ELM can quickly detect cadmium in rice stems, and accurately distinguish different degree of cadmium pollution. To complete the evaluation of LIBS on the ability of quantitative detection of cadmium in rice stems, multivariate analysis methods such as PLSR, LS-SVM, RBFNN, and ELM were used for modeling. The LIBS spectra ranged from 210.01 to 231.00 nm and contained 1,024 variables. It can be found that, the ELM model had the best correlation coefficient and lowest prediction error, with *R*_*p*_ of 0.995 and RMSEP of 28.96 mg/kg in prediction set. It indicated that multivariate analysis with ELM method can realize the fast and accurate detection of cadmium content in stem more effectively. Compared with traditional detection methods (more than 150 min), the combination of LIBS technology and ELM method (less than 5 min) greatly reduces the time required to detect heavy metals on a sample. The results show that the combination of LIBS technology and chemometrics provides significant advantages for fast and accurate detection of cadmium contamination degree and cadmium content in rice stems. The method can timely diagnose the straw containing cadmium, prevent the risk of heavy metals in the straw, ensure the safety of the straw as a clean energy, and improve utilization rate of energy.

## Data Availability Statement

The raw data supporting the conclusions of this article will be made available by the authors, without undue reservation.

## Author Contributions

WW, WK, and FL conceived and designed the experiments. WW, TS, ZM, and YL performed the experiments. WW, TS, WZ, and YL contributed to data analysis. YH, FL, and YL contributed to reagents, materials, and analysis tools. WW, TS, and FL wrote the whole manuscript. All authors contributed to manuscript revision, read, and approved the submitted version.

## Conflict of Interest

The authors declare that the research was conducted in the absence of any commercial or financial relationships that could be construed as a potential conflict of interest.
